# Identification and characterization of two new markers for differentiating fall armyworm strains across the Western Hemisphere

**DOI:** 10.1371/journal.pone.0350388

**Published:** 2026-06-02

**Authors:** Rodney N. Nagoshi, Ashley E. Tessnow, Robert L. Meagher

**Affiliations:** 1 Center for Medical, Agricultural and Veterinary Entomology, United States Department of Agriculture-Agricultural Research Service, Gainesville, Florida, United States of America; 2 Oak Ridge Institute for Science and Education, Oak Ridge Associated Universities, Department of Energy, Oak Ridge, Tennessee, United States of America; Lavras Federal University, BRAZIL

## Abstract

The fall armyworm (FAW) (*Spodoptera frugiperda* (J.E. Smith)) is a major pest of corn and several other crops. Although native to the Western Hemisphere, established populations were detected in western Africa in 2016 and have since been found in most corn producing areas in the Eastern Hemisphere. FAW consists of two populations historically called “host strains” that are believed to be undergoing sympatric speciation with reproductive isolation being driven primarily by host plant use. The C- and R-strains are morphologically indistinguishable and so can only be identified by a small set of molecular markers with polymorphisms in a single locus, the Triosephosphate isomerase gene (*Tpi*), currently the most commonly used for population studies. This reliance on a single marker limits confidence in the accuracy of strain identification and the consequent extrapolations of strain characteristics, indicating a need for additional markers to test these findings. This paper describes two new strain markers that provide new genetic tools for strain analysis. The data provide support for earlier conclusions about the primacy of the *Z*-chromosome in strain identity, the strain divergence of the *Z*-chromosome as a single genetic unit and expand upon previous observations to quantify differences in how each strain is evolving. These markers will facilitate further studies on the population behavior of FAW in the Western Hemisphere and should advance our understanding of the global movements of this important agricultural pest.

## Introduction

Sympatric speciation describes a process by which two populations capable of interbreeding and occupying overlapping geographical ranges can still sustain genetic divergence [1]. One mechanism by which this could occur is through differences in host plant use that causes sufficient reproductive isolation to significantly curtail gene flow [2]. Such “host races” have long been hypothesized as representing an intermediate speciation stage, initially allowing enough divergence for the evolution of biological reproductive incompatibility that defines complete speciation [3].

One Lepidopteran species hypothesized to be undergoing this process is the fall armyworm (FAW) (*Spodoptera frugiperda* (J.E. Smith)) [4], a significant agricultural pest with a broad host range. FAW is reported to feed on several hundred plant types, though major infestations in the field have only consistently been observed in a few crop species with corn suffering the greatest economic impact [5,6]. FAW is native to the Western Hemisphere where it is comprised of two populations that appear to be analogous to host races (reviewed in [7]). These were historically designated as “host strains” and named for the crop on which they were originally identified, with the corn strain associated with corn and sorghum while the rice strain predominates in pasture, turf, and forage grass species [8,9]. Despite its initial description, subsequent studies demonstrated that rice is probably a secondary host with inconsistent host specificity [10–13]. To avoid potentially exaggerating the risk to rice agriculture, we have taken to calling the corn and rice strains as C-strain and R-strain, respectively. Although these populations are considered host strains, their host association is not absolute. The R-strain is regularly found in corn and sorghum, especially in the Northern Hemisphere, while the C-strain is infrequently found in pasture, turf, and forage.

The host strains are morphologically indistinguishable, leaving only molecular markers as a means of identification [4,14]. Most useful to population studies that require large numbers of individual sampling are genetic markers that can be assessed from single specimens using DNA amplification (PCR) methods. These have been limited to mitochondrial haplotypes, particularly those defined by segments of the *Cytochrome oxidase I (COI)* gene and polymorphisms in the *Z*-chromosome linked to the *Tpi* gene that encodes the housekeeping function Triosephosphate isomerase, with the latter showing stronger and more consistent correspondences with host plant use [7,15]. The limited number of strain markers raises concerns about the accuracy of strain identification, particularly since the mitochondrial markers do not appear to identify strains in populations recently detected in the Eastern Hemisphere [13]. This leaves *Tpi* as the marker most documented for strain identification in current studies.

Whole genome sequencing (WGS) techniques have recently been applied to strain identification with mixed results [16–25]. Several studies failed to find distinctive WGS SNP groupings consistent with the host strains [16,19–21,23]. In three studies where strain-specific WGS SNP groups were found, the preponderance of these SNPs mapped to the *Z*-chromosome, suggesting an outsized role for this chromosome in strain divergence [18,22,24].

In contrast, the role of autosomal functions in strain differentiation is uncertain. WGS analyses have identified a small number of autosomal candidates, but these appear to differ between studies (compare [18,22,24]). The autosomes when analyzed as a group do show significant differentiation between the strains but at a substantially lower level than exhibited by the *Z*-chromosome [22]. While this is consistent with strain divergence at the autosomal level, the dearth of autosomal sites with reproducible strain-specificity suggests that this divergence is likely to be stochastic. One potential explanation is strain divergence being driven by *Z*-linked functions, with incidences of autosomal strain-specificity occurring by chance, *i.e.*, genetic hitchhiking. In this scenario strain-specific autosomal loci will vary by collection and the effectiveness of WGS approaches in identifying the strains will depend on the representation of *Z*-linked SNPs in the analysis.

The suggested centrality of the *Z*-chromosome, where males are *ZZ* and females are *ZW*, in FAW strain divergence is consistent with observations that the sex chromosome evolves more rapidly than autosomes, a pattern known as the “Fast-*Z*” (or Fast-*X*) effect [26]. This is mainly due to hemizygosity in the heterogametic sex (females in Lepidoptera), which allows recessive alleles to be expressed and selected, and to reduced effective population size that enhances genetic drift [27,28]. Examples of *Z*-chromosome driven divergence in Lepidoptera include host strain divergence in *Ostrinia* moths [29], species differentiation in *Colias* butterflies [30], and lineage divergence in *Morpho* butterflies [31]. These represent multiple precedents for the hypothesis that FAW strain divergence is being driven primarily, if not solely, by *Z*-linked genes.

A genetic understanding of the FAW strains will provide tools to elucidate their ecological and evolutionary dynamics thereby improving pest management strategies. The strains have been reported to differ in their mating behaviors, host use, immune responses, and pesticide resistance profiles [8,32–39]. Additional and improved strain markers will facilitate the confirmation of these phenotypic differences and their exploitation to develop targeted control measures. Additional strain markers are particularly relevant for studies on the Eastern Hemisphere, where the recent (2016) discovery of established FAW populations in Africa and its subsequent detection in most corn-producing nations in the hemisphere raise concerns about what crops are at risk and where permanent populations will become established [40].

The objectives of this study are to identify new strain markers to complement *Tpi* and in doing so test aspects of strain divergence suggested by *Tpi* phylogenetic studies [41]. The accuracy of the markers is measured by genetic and phylogenetic analyses of DNA sequences from field-collected larvae obtained from multiple locations and times in the two Americas, with validation based on statistically significant correspondences with host plant use and the *Tpi* marker. The phylogenetic patterns of the new markers are compared with that previously described by *Tpi* to test assumptions about the relationships between the two strains and differences in their evolutionary pathways.

## Methods

### Identification of Z-linked genes

WGS data is previously described and consists of 220 C-strain and 183 R-strain moths collected across the continental United States and Puerto Rico [22] (NCBI SRA BioProject ID PRJNA1115088). To evaluate patterns of strain specificity on the *Z*-chromosome across the native range, including South and Central America, regions for which we did not have sufficient DNA for whole-genome sequencing, we identified six genes on the *Z*-chromosome (including *Tpi*) that exhibited within strain variability and that could be used to develop a PCR based screening assay. To do this, we first extracted 472 *Z*-chromosome genes with annotated functions from the published OGS6.1 genome annotation (https://bipaa.genouest.org/sp/spodoptera_frugiperda_pub/download/annotation/corn/OGS6.1/) that corresponded with our reference genome. Using the Unix global recognition expression print (grep) search command we further refined the genes listed in our annotation file to include only those that had been previously annotated in an insect species, excluding those annotated in vertebrates such as humans, frogs, and mice. This resulted in 149 candidate genes. To facilitate the PCR isolation and DNA sequence analysis of both exon and intron segments, we focused on genes that were between 2,000 and 7,000 base pairs in length and contained 2–5 introns. In addition, to explore the extant of the Z-chromosome important to strain identity and to possibly gain insight into the number and distribution of strain-defining functions, potential marker loci were selected that were physically distant (>1 Mb) from each other and *Tpi* so that there would be a reasonable expectation of crossing over (recombination) between markers at frequencies high enough to be detected by the sample sizes in this study.

To ensure strain variability we used VCFtools to split a *Z*-chromosome SNP VCF file, derived from our WGS data into two subsets: one containing C-strain variants and the other containing R-strain variants [42]. Each subset was further filtered with VCFtools to retain only biallelic loci (minimum and maximum alleles = 2) with a minor allele frequency (MAF) within the strain of ≥ 0.05. We then mapped the filtered VCF files and GFF3 annotation onto the fall armyworm genome using IGV Viewer v. 2.19.1 [43], from which we selected six candidate genes for further analysis.

Initial PCR experiments identified two loci that were successfully amplified and characterized. The *UBC4* gene is associated with an mRNA sequence (Genbank: XM035577613) predicted to encode for the FAW ubiquitin-conjugating enzyme E2-22. The XM035577613 sequence was aligned with the FAW isolate SF20−4 chromosome 25 whole genome shotgun sequence released by the Australian Pest Genome Partnership (NC064236) to identify the intron within the XM035577613 transcribed segment and to map the intron/exon boundaries. The second loci designated *nes* is located on the opposite side of *Tpi* from *UBC4* and is associated with an mRNA sequence (XM035581013) predicted to encode for lysophospholipid acyltransferase 5. Alignment of the 5’ half of this sequence with NC064236 was used to extrapolate the exon/intron structure. The *Tpi* gene exon/intron structure was previously characterized [15].

Sample Collection and DNA Isolation for the analysis of *UBC4*, *nes*, and *Tpi*.

Larval collections comprising a total of 611 specimens were made from both C-strain and R-strain associated plant hosts from multiple times and locations in North America (NA) and South America (SA, Table 1). Subsets of the Florida, Argentina, and Brazil specimens were previously used for a phylogenetic analysis of the *Tpi* marker [41]. In this study, a total of 207 specimens from Mississippi (96), Texas (96) and Paraguay (15) were added.

**Table 1:** Description of larval collections from C-strain (corn, sorghum, cotton) and R-strain (turf grasses, pasture grasses, rice, millet, alfalfa) associated host plants use in the amplification of the identified *Z*-chromosome strain markers.

State/Country	County/Province	Collection date	Host plant	n	Collector
Florida	Highlands	Oct 2003	C-strain	27	R. Meagher
Florida	Palm Beach	Oct 2003	C-strain	57	R. Meagher
Florida	Miami-Dade	Jan 2005	C-strain	14	R. Meagher
Florida	Miami-Dade	Oct 2003	R-strain	10	R. Meagher
Florida	Alachua	Sep 2003	C-strain	10	R. Meagher
Florida	Hardee	Sep 2002	R-strain	24	R. Meagher
Mississippi	Washington	Jul-Aug 2006−7	C-strain	13	C. Davies
Mississippi	Washington	Jul-Aug 2006, 2008	R-strain	42	C. Davies
Mississippi	Hinds	Sep 2007	R-strain	6	C. Davies
Mississippi	Oktibbeha	Aug 2007	C-strain	35	C. Davies
Texas	Brazos	Jul, Sep 2004	C-strain	96	J. Lopez
Argentina	Corrientes	2011	R-strain	28	M.G. Muruá
Argentina	Salta	Feb 2012	C-strain	20	M.G. Muruá
Argentina	Tucumán	Feb 2012	R-strain	40	M.G. Muruá
Argentina	Buenos Aires	Feb 2012	R-strain	33	M.G. Muruá
Argentina	Cordoba	Feb 2012	R-strain	40	M.G. Muruá
Brazil	Mato Grosso	2005	C-strain	76	P. Silvie
Brazil	Mato Grosso	2005	R-strain	25	P. Silvie
Paraguay	Caacupé	2005	C-strain	15	P. Silvie

Specimens were archived at −20°C until use for DNA isolation. Genomic DNA was isolated from single specimens by homogenization in a 5-ml Dounce homogenizer (Thermo Fisher Scientific, Waltham, Massachusetts, USA) in 1 ml of phosphate buffered saline (PBS, 20 mM sodium phosphate, 150 mM NaCl, pH 8.0). The homogenate was transferred to a 2-ml microcentrifuge tube and pelleted by centrifugation at 6000 g for 5 minutes at room temperature. The pellet was resuspended in 400 µl Genomic Lysis buffer (Zymo Research, Orange, California, USA) and incubated at 55°C in a dry bead bath for at least three hours. Debris was removed by centrifugation at 10,000 rpm for 5 minutes. The supernatant was transferred to a Zymo-Spin II or Zymo-Spin III column (Zymo Research, Orange, California, USA) and processed according to manufacturer’s instructions.

### PCR method and sanger sequencing

A variation of nested PCR was used to isolate relevant segments from the *UBC4*, *Tpi*, and *nes* genes. The standard protocol had a first PCR reaction using the outer most primers (Fig 2) in a 30-µl reaction mix containing 3 µl of 10X manufacturer’s reaction buffer, 0.5 µl 10mM dNTP, 0.5 µl 20-µM primer mix, 1 µl DNA template (between 0.05–0.5 µg), 0.5 units Taq DNA polymerase (New England Biolabs, Beverly, Massachusetts) with the remaining volume in water. The thermocycling program was 94°C (1 min), followed by 25 cycles of 92°C (30 s), 56°C (45 s), 72°C (45 s), and a final segment of 72°C for 3 min. The completed reactions were diluted with 50 µl water and 1 µl was used for the 2^nd^ PCR reaction using the same protocol. The PCR products were separated by agarose gel electrophoresis and purified using the Zymoclean Gel DNA Recovery Kit (Zymo Research, Orange, California). All PCR reactions were performed in 96-well PCR plates (Thermo Fisher Scientific, Waltham, Massachusetts, USA). The PCR products were separated by agarose gel electrophoresis and purified using the Zymoclean Gel DNA Recovery Kit (Zymo Research, Orange, California). The isolated fragments were directly analyzed by DNA sequencing performed by Azenta Life Sciences (Chelmsford, Massachusetts).

Primers used for the PCR amplification of *UBC4* were, 1^st^ reaction u095F (5’- TTAACGACAGCTGGACGGAG −3’) and u590R (5’-GAGAGGGCCACAGAACATCT-3’) and 2^nd^ reaction u138F (5’-ACCGGACACACCATACGAAG-3’) and u557R (5’-AGGGTAGCGCTTTCTACGTT-3’). Primers for the PCR amplification of *nes* were 1^st^ reaction n3085F (5’-GGGAGTCATAGGGGCAACTG-3’) and n3970R (5’-TTTCCTGCTGGCTACTCACC-3’) and 2^nd^ reaction n3212F (5’-AGCAACGGGGTTAGACATGG-3’) and n3907R (5’-TCGGACGAGTTCAGGGAAAC-3’). Primers for the PCR amplification of *Tpi* were 1^st^ reaction t282F (5’- GGTGAAATCTCCCCTGCTATG-3’) and t1195R (5’-CAGTATGGTGGGTCAGTGACT −3’) and 2^nd^ reaction t412F (5’- CCGGACTGAAGGTTATCGCTTG −3’) and t1140R (5’- GGTTGTCAGCGAATGCTTCCGC-3’). All primers were synthesized by Integrated DNA Technologies (Coralville, Iowa).

### *Z*-linked marker assessment and analysis

**Fig 1:**
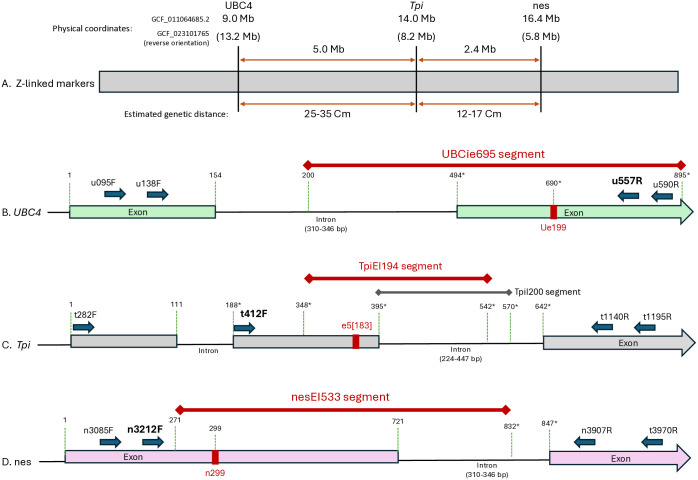
Molecular map of relevant regions of the three *Z*-linked loci used for strain identification. A, map of Z-chromosome showing approximated locations of *UBC4*, *Tpi*, and *nes* and their physical and estimated genetic distances. Physical coordinates are from two whole genome assemblies with the listed RefSeq Assembly Accession Number listed. The assemblies report the Z-chromosome (chromosome 1 for GCF_011064685.2 and chromosome 25 for GCF_023101765.2) in opposite orientations but the distances between the genes are the same. Estimated recombination frequencies between loci (in centimorgans, Cm) are extrapolated as explained in the text. B, C, and D, are molecular maps of the relevant segments of *UBC4*, *Tpi*, and *nes,* respectively. In each case, two exons (boxes) and the intervening intron (lines) are used for PCR amplification with the primers noted by larger font and in bold also used for DNA sequencing. The intron segments are of variable length because of frequent indels. As a result, the length coordinates after an intron are approximate as indicated by an asterisk. The blue block arrows approximate the locations of primers used for PCR amplification and DNA sequencing. The red vertical bars identify for each gene a SNP that shows strain-specificity. The red horizontal lines identify the DNA segment for each locus that is used for the phylogenetic analysis. The TpiI200 segment was previously used for the initial *Tpi* phylogenetic study [41].

The *UBC4*, *Tpi*, and *nes* genes are located on the *Z*-chromosome with physical distances and gene order determined by the ZJU_Sfru_1.1 (GCF_011064685.2) and confirmed by the AGI-APGP_CSIRO_Sfru_2.0 (GCF_023101765.2) whole genome assemblies that are available at NIH-NCBI, https://www.ncbi.nlm.nih.gov/gdv/browser/genome/?id=GCF_011064685.2 (Fig 1A). Segments from each of these loci were identified that contained a portion of both one exon and an adjacent intron (Fig 1B-D). SNPs from the less variable exon segment can be potentially used as an easily detected strain marker while the intron segment provides higher genetic variation that increases the resolution of the phylogenetic analysis. The genetic studies are complicated by the occurrence of SNPs and indels that when heterozygous (in *ZZ* males) will produce overlapping DNA sequence chromatographs that provide ambiguous sequence information. These polymorphisms are most frequent in the highly variable intron regions. To optimize the number of specimens that can be used in the phylogenetic studies, the intron portions of the marker segments were truncated as needed at either at the 5’ (*UBC4*) or 3’ (*Tpi, nes*) ends. The final exon/intron sequence used is a continuous segment of the marker gene that strikes a balance between providing genetic variation while reducing the frequency of unusable ambiguous events.

Phylogenetic analyses were previously performed for the *Tpi* marker using the TpiI200 intron segment (Fig 1C). In this study, the *Tpi* analysis was repeated using the TpiEI194 segment that differs from TpiI200 by the inclusion of 47 bp of exon sequence at the 5’ end and deletion of 28 bp of intron sequence at the 3’ end. The latter removed a polymorphic SNP that produced a high number of problematic heterozygotes, thereby increasing the number of useable sequences.

SNPs were identified in exons for each of the three markers that were empirically shown to correspond with the strain-defining phylogenetic groupings (red vertical lines, Fig 1B-D). We note that identification of these strain-specific exon SNPs was not impacted by lesions in the intron, provided DNA sequencing was initiated from the promoter on the same exon as the SNP marker. This avoided the consequences of frameshift mutations most often found in introns that causes misaligned downstream sequencing. Specifically, the u557R primer for the UBCie695 segment to detect Ue199 (Fig 1B), t412F for the TpiEI194 segment to detect e5[183] (Fig 1C), and n3212F for the nesEI533 segment to detect n299 (Fig 1D).

### Sample sizes and heterozygosity

PCR amplification and DNA sequence analysis for all three markers were performed on the 611 specimens described in Table 1. The number of useable sequences varied by marker because of differences in amplification efficiency and the frequency of heterozygotes. The latter results from male specimens having two *Z*-chromosomes with the potential for DNA sequence heterozygosity. These will exhibit ambiguous sequence information at the mismatched sites, and it is especially problematic in the more variable intron segments where frameshift mutations are common. Only unambiguous sequences were used in the analyses with the following sample sizes, UBCie695 (North America or NA = 152, South America or SA = 80), TpiE194 (NA = 241, SA = 190), and nesEI533 (NA = 226, SA = 189). These represented specimens either homozygous for the relevant DNA segment (males) or hemizygous for the Z-chromosome (females). Because all specimens were analyzed by the same methodology, we believe it unlikely that this exclusion of heterozygotes should bias the comparisons between strains.

### Strain identification

Three related methods are used to determine the strain identity of a specimen depending on the analysis. One is based on the host plant from which the specimen was collected. The second is derived from phylogenetic groupings that correlate with strain-specific host plant use. The third is defined by exon SNPs that correlate with the strain-specific phylogenetic groups. These are expected to be generally in agreement and the method used is specified when relevant.

### Phylogenetic analysis

Phylogenetic trees were produced with Geneious Prime 2021.1.1 software [44] using both the Neighbor-Joining (NJ) and the Maximum Likelihood (ML) [45] methods. The ML analyses was subjected to bootstrap testing (1000 replicates) using the TN93 nucleotide substitution model [45], with the optimal tree shown and drawn to scale. The evolutionary distances were computed using the Maximum Composite Likelihood method [46]. Nucleotide diversity and haplotype diversity analyses were performed using DnaSP [47].

### Analysis and presentation of data

DNA sequence analyses including alignments and phylogeny were performed using Geneious Prime 2021.1.1 (Biomatters, Auckland, New Zealand). The generation of graphs were done using Excel and PowerPoint (Microsoft, Redmond, WA). Statistical analysis including ANOVA and t-tests were performed using the open-source JASP program [48]. The DNA sequences for UBCie695, TpiEI194, and nesEI533 are describe in supplementary Figure S1 Fig. Sequences used in the phylogenetic analysis are deposited into GenBank: UBCie695 (PX681553-PX681786), TpiEI194 (PX681217-PX681552), nesEI533 (PX680873-PX681216). These sequences can be accessed from the National Library of Medicine, (https://www.ncbi.nlm.nih.gov/genbank/). Geographical maps were generated using QGIS version 2.18.2 (Open Source Geospatial Foundation).

## Results

### Identification of new strain-specific markers on the Z-chromosome

The WGS SNP data allowed identification of two *Z*-linked loci that can differentiate the two FAW host strains. The *UBC4* and *nes* loci lie on either side of *Tpi* and together span approximately 1/3 the physical length of the *Z*-chromosome, an approximately 7.4 Mb segment (Fig 1a). Reported recombination frequencies in Lepidoptera have ranged from a mean of 5–8 Cm/Mb for the whole genome [49–52], with a recent estimate of mean recombination frequency on a butterfly Z-chromosome of 7.03 cM/Mb [50]. Based on these data, we estimate an expected recombination frequency between *UBC4* and *nes* of about 37% to 50%, which should be detectable within the sample sizes used in this study.

Segments from the *UBC4*, *Tpi*, and *nes* loci were identified that contained a portion of both one exon and an adjacent intron. The UBCiE24 (Fig 1B), TpiEI194 (Fig 1C), and nesEI533 (Fig 1D) segments were examined from specimens collected from either C-strain or R-strain associated host plants. In this technique, strain specificity is indicated by the asymmetric association of host plant usage with specific phylogenetic clusters as previously demonstrated for an intron fragment from *Tpi* [41].

**Fig 2:**
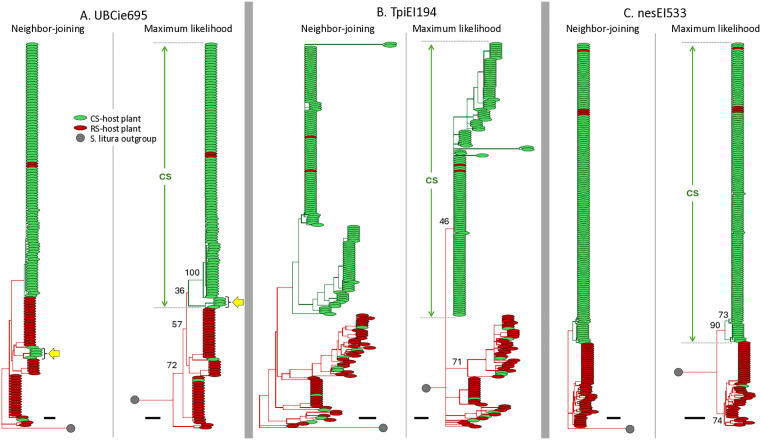
Phylogenetic analysis of North American FAW collections. Neighbor-Joining trees were initially constructed, followed by Maximum-Likelihood analysis with 1000X bootstrapping for the same set of sequences. Selected bootstrap values are shown at branch points. Each sequence is color-coded for the strain category of the host plant from which the sample was collected. Scale bar represents 0.02 substitutions per site. Yellow arrows identify sequences that were placed in different strain-specific groups by the two phylogenetic methods. The branch length of outgroup (grey circle) is not drawn to scale.

**Fig 3:**
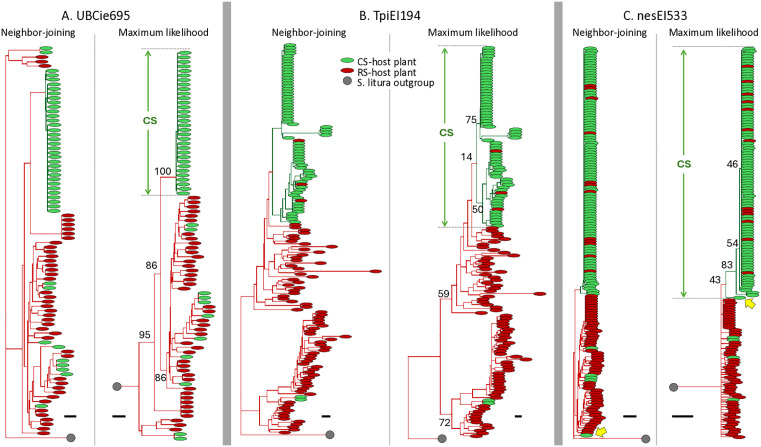
Phylogenetic analysis of the South America FAW collections. Analysis as described in Fig 2.

The three DNA segments were each analyzed by Neighbor-Joining (NJ) and Maximum-Likelihood (ML) phylogeny programs for collections from multiple locations in North America (NA) and South America (SA), resulting in a total of 12 phylogenetic trees. In the NA collections a phylogenetic cluster predominantly associated with C-strain host use was consistently observed arising from a single branchpoint (ancestor) in all trees (Fig 2). The remaining specimens were primarily found in R-strain host plants and usually in a more dispersed pattern with indications of multiple phylogenetic lineages. A similar result was observed with the SA collections (Fig 3). Again, a clade can be identified in each tree that is predominated by and contains most of the specimens collected from C-strain host plants.

There were two cases where differences were observed in the composition of the C-strain and R-strain groupings between the NJ and ML trees. One involved four specimens consisting of two haplotypes in the North America UBCIe695 analyses (yellow arrow, Fig 2A) and the other a single specimen in the South America nesEI533 analyses (yellow arrow, Fig 3C). Bootstrap support for the TpiEI194 C-strain clade was frequently weak (<40), particularly for the TpiEI194-derived C-strain clade, indicating in these cases that the details of the branching patterns are uncertain. However, the consistent observation of a C-strain host predominated clade in both NJ and ML trees from both collections and with all three genetic markers supports the existence a discrete C-strain phylogenetic group.

**Fig 4:**
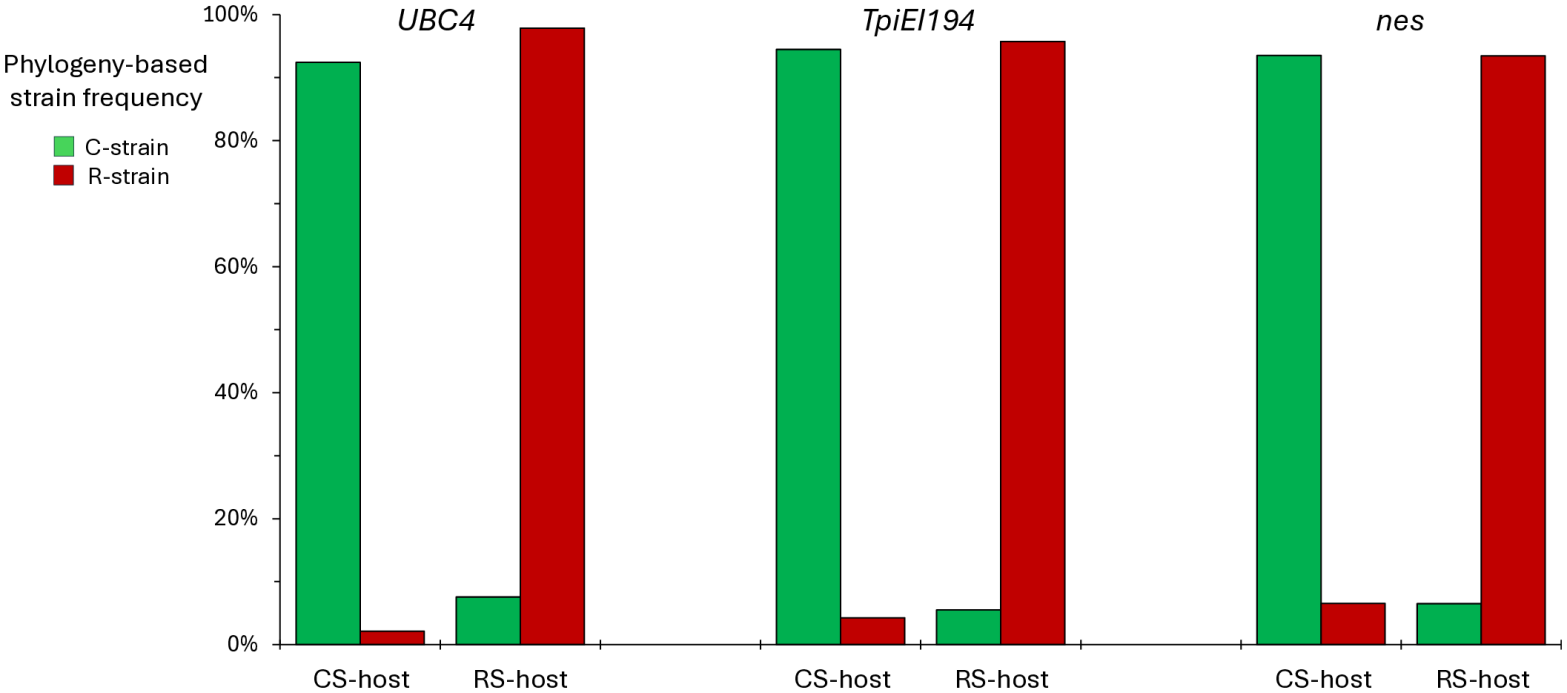
Correspondence between phylogeny-based strain identification and host plant. The graph displays the frequency that each strain as identified by phylogeny was found on strain-associated host plants.

Because similar results were obtained for both the NA and SA collections, data from the two continents were pooled to quantify the association with host plant use, the defining phenotype of the host strains. Greater than 90% of the combined C-strain clade was associated with C-strain host plants (Fig 4). Similarly, the remaining specimens were greater than 90% associated with R-strain host plants.

### Association of the phylogeny-defined strain identity with SNPs

**Fig 5:**
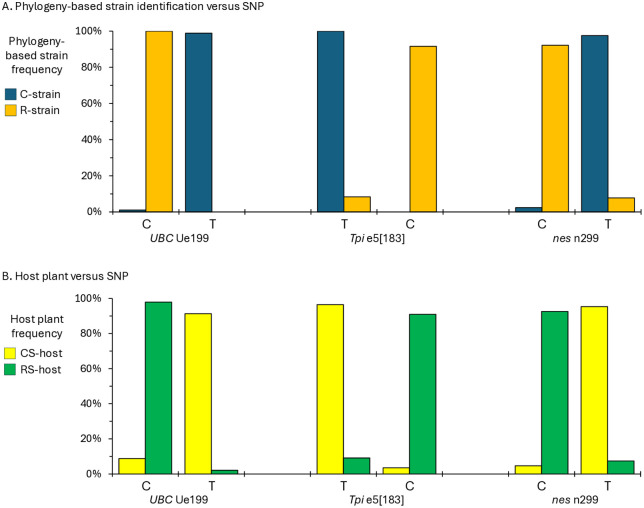
Association of *UBC*, *Tpi*, and *nes* SNPs with strain-specific phylogenetic groupings and host plants. A, frequency of strains as identified by phylogenetic groupings per each strain-specific SNP. B, frequency of strains as identified by host plant use per strain-specific SNP.

To facilitate strain detection, we examined the association of specific SNPs with host plants and phylogeny, using the previously characterized *Tpi* e5[183] SNP as a model [53]. The strain-specific phylogenetic groupings derived from the ML derived trees from each location were pooled. The C-strain and R-strain groups derived using the UBCie695 phylogeny showed 100% correspondence with a cytosine (C) at site Ue199 (Ue199-C) compared to 99% association with Thymine (T, Ue199-T), respectively (Fig 5A). The *nes* phylogenetic groups are similarly linked to polymorphisms at *nes* site n299, with 95% of the C-strain nes clade n299-T and 93% of the R-strain clade n299-C. Both the Ue199 and n299 SNPs show the same degree of linkage with the strain-specific phylogenetic groups as *Tpi* e5[183]. A similar degree of correspondence was found between the *UBC* Ue199, *nes* n299, and *Tpi* e5[183] SNPs with strain-specific host plant use (Fig 5B).

### Examination of within strain genetic variation

**Fig 6:**
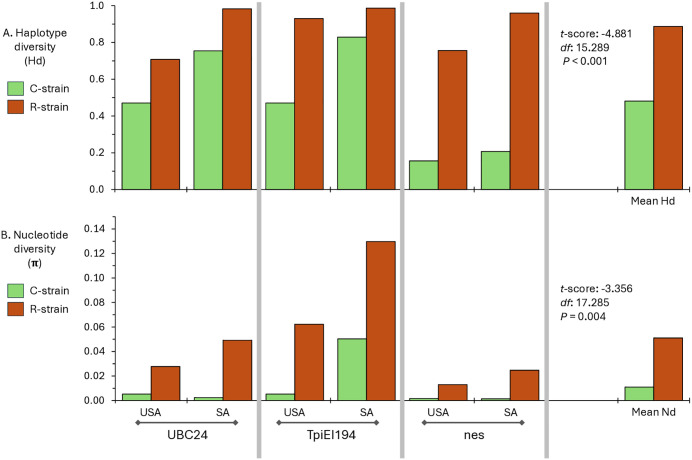
Examination of genetic variation within the strain populations as defined by the Maximum-Likelihood phylogenetic analysis. The Y-axis shows the Hd (A) and π (B) values exhibited by the two strain populations (color-coded) as defined by each of the three marker loci. The results of collections from the two Americas are displayed separately. At the far right are the mean values of the three markers and results of two-tailed *t*-test comparisons of the genetic variation metrics for the two strains.

The strain-associated phylogenetic groups from each of the trees in Figs 2 and 3 were separately analyzed for nucleotide diversity (π) and haplotype diversity (Hd), with the means for each strain compared (Fig 6). The C-strain specimens displayed significantly less variation by these two metrics than the R-strain regardless of location or phylogenetic method. When combined across all three genes and both regions, haplotype diversity of the C-strain is 0.48 ± 0.27 (mean ± s.d.) compared to 0.89 ± 0.12 for the R-strain (Fig 6A). Similarly, the C-strain nucleotide diversity is substantially lower than the R-strain, 0.011 ± 0.019 versus 0.051 ± 0.042 (Fig 6B).

### Measurements of divergence between populations

The divergence of the two strains can be quantified using Wright’s fixation index (*Fst*) as was previously demonstrated for the *Tpi* marker [41]. We repeated the *Fst* analysis of *Tpi* with the current collection and applied it to sequences from UBCie695 and nesEI533. In addition, absolute divergence (*Dxy*) was calculated for all three loci (Table 2).

**Table 2:** Genetic differentiation between populations as measured by *Fst* and absolute divergence (*Dxy*). Abbreviations: NA, North America; SA, South America; CS, C-strain host; RS, R-strain host.

		Population-1		Population-2			
	Marker	host plant	Continent	host plant	Continent	*Fst*	*Dxy*
U1	UBCie695	CS	NA	RS	NA	0.548	0.051
U2	UBCie695	CS	NA	RS	SA	0.509	0.056
U3	UBCie695	RS	NA	CS	SA	0.338	0.050
U4	UBCie695	CS	SA	RS	SA	0.310	0.054
U5	UBCie695	CS	NA	CS	SA	0.073	0.019
U6	UBCie695	RS	NA	RS	SA	0.110	0.049
T1	TpiEI194	CS	NA	RS	NA	0.559	0.126
T2	TpiEI194	CS	NA	RS	SA	0.391	0.113
T3	TpiEI194	RS	NA	CS	SA	0.572	0.130
T4	TpiEI194	CS	SA	RS	SA	0.407	0.116
T5	TpiEI194	CS	NA	CS	SA	−0.004	0.031
T6	TpiEI194	RS	NA	RS	SA	0.090	0.103
n1	nesEI533	CS	NA	RS	NA	0.682	0.027
n2	nesEI533	CS	NA	RS	SA	0.531	0.026
n3	nesEI533	RS	NA	CS	SA	0.645	0.027
n4	nesEI533	CS	SA	RS	SA	0.495	0.026
n5	nesEI533	CS	NA	CS	SA	0.010	0.003
n6	nesEI533	RS	NA	RS	SA	0.112	0.021
					Mean	0.354	0.057

*Fst* measures how much the genetic differences between populations accounts for the total variation of the populations. This makes it a relative measure of differentiation that is influenced by both genetic differences between the populations as well variations in within population diversity. Higher values (approaching 1.0) indicate greater divergence and lower gene flow between populations. The *Dxy* metric describes the genetic differences between groups and is not strongly affected by within population variation. Therefore, this metric provides an absolute measurement of divergence between groups. A total of 18 comparisons were made, six for each marker. The mean *Fst* was 0.354 and the mean *Dxy* 0.057 (Table 2).

### Comparisons between the *UBC4*, *Tpi*, and *nes* loci

**Fig 7:**
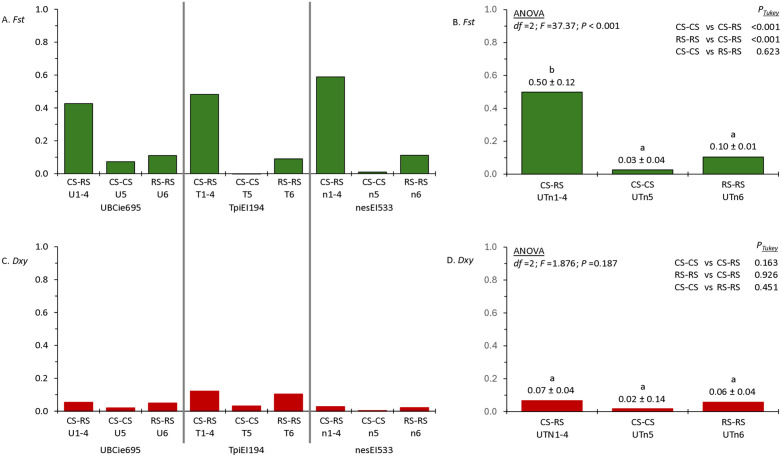
Calculations of *Fst* and *Dxy* for CS-RS comparisons with ANOVA analysis to identify differences between genetic markers. A, *Fst* analysis for the UBCie695, TpiEI194, and nesEI533 segments. The CS-RS columns represent the mean of the four CS-RS comparisons done for each segment. The values are from Table 2 as indicated by labels below the columns. B, the data from A combined and analyzed by ANOVA with Tukey Post Hoc testing. The mean ± sd is given above the columns with different lower-case letters indicating statistical significance at *p* < 0.05 level. C, *Dxy* analysis for the UBCie695, TpiEI194, and nesEI533 segments. D, the data from C combined and analyzed by ANOVA with Tukey Post Hoc testing.

*Fst* and *Dxy* comparisons between CS and RS populations were made for each marker loci. For each marker, the mean *Fst* for between strain comparisons (CS-RS) was always substantially higher than those calculated for within strain (CS-CS and RS-RS) comparisons and the CS-CS *Fst* was consistently lower than that of RS-RS (Fig 7A). Statistical analysis by ANOVA demonstrated that the between strain to within strain differences were statistically significant while those between CS-CS and RS-RS were not (Fig 7B).

In contrast, *Dxy* values were generally low (<0.14) for all comparisons and marker loci (Fig 7C). For all three tested marker segments the *Dxy* value for CS-RS was like that of RS-RS and both were higher than CS-CS comparisons. However, none of these differences were significantly different (Fig 7D). These results indicate that for the three *Z*-linked marker segments, divergence between the strains is occurring by mechanisms that increase *Fst* much more than *Dxy*.

### Comparisons between strains and continental populations

**Fig 8:**
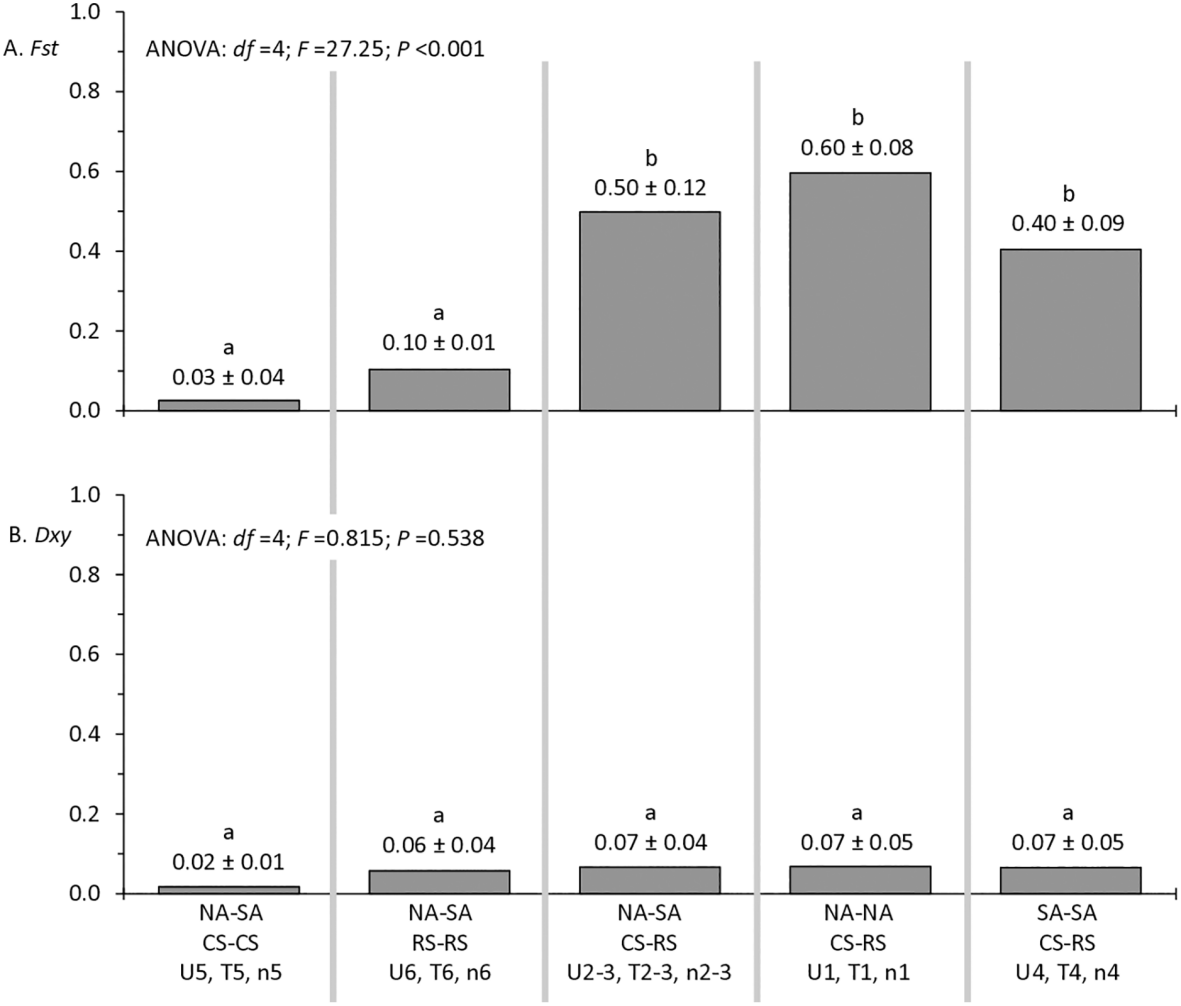
Calculations of *Fst* and *Dxy* categorized to show the relative contributions of strain identity and geographical separation to these metrics. The data from Table 2 were arranged to compare populations within and between the two American continents (NA = North America, SA = South America) in combination with strain identity as determined by phylogeny. A, *Fst* measurements comparing populations with different combinations of strain identity and continental origin. Mean values ± standard deviations are shown above columns. Means with different letters are significantly different as calculated by Tukey Post Hoc testing. B, same as A but with *Dxy*. Codes shown below the column indicate the data used from Table 2.

The collection of FAW specimens from two continents and different time periods provides an opportunity to measure the genetic divergence of populations separated by geography and time. Low *Fst* values (≤ 0.1) were observed for both within strain comparisons (CS-CS and RS-RS) despite each involving comparisons between the widely separated NA and SA populations (Fig 8A). Similarly, the much higher *Fst* values in all the CS-RS comparisons showed no statistically significant differences in the results from between continental (NA-SA) and within continental (NA-NA and SA-SA) populations. Consistent with our past results, the *Dxy* calculations were consistently low (< 0.1) for all comparisons regardless of strain and location, with no significant differences found (Fig 8B). Taken together, there appears to be no significant population divergence associated with geographical or temporal separation with respect to our *Z*-linked markers. Population divergence within the survey indicates FAW populations appear primarily, if not solely, due to strain.

## Discussion

Genetic studies on the FAW host strains have been hindered by the small numbers of markers demonstrated to identify the strains in both North and South American populations. We believe testing using FAW from both continents is essential for the validation of a strain marker because of multiple examples of characteristics that exhibited strain specificity limited to geographical regions, laboratory colonies, or could not otherwise be replicated. These include female pheromone composition [54,55], time of mating [56,57], and wing size [58,59]. Such incidences of variable and transient strain specific phenotypes are to be expected in a highly mobile species where reproductive isolation between the strains is dependent on environmental factors like wind vectors and host plant distribution. These conditions create the potential for significant differences in gene flow between the strains that vary by region and season. In addition, inconsistencies in the choice and use of presumptive strain-specific molecular markers could result in the misidentification of the strains in one or more of these studies. For these reasons, we believe that the validation of molecular markers claimed to be strain specific requires sampling over multiple time periods and at multiple locations on both American continents. Prior to this study, only mitochondrial haplotypes and the *Z*-linked *Tpi* gene had met these criteria, with the former recently shown to no longer be strain specific in Eastern Hemisphere populations [13]. As a result, much of what we assume genetically about the host strains stems only from analyses based on the *Tpi* gene, which while suggestive elicits limited confidence without confirmation by other methods.

The strain specificity exhibited by polymorphisms in the *UBC4* and *nes* loci allow testing conclusions about strain evolution previously based solely on the phylogenetic analysis of a portion of a *Tpi* intron [41]. The rationale of the *Tpi* study was that its tight linkage to one or more strain-defining functions justifies its use as a proxy for the phylogenetic description of strain divergence. The agreement of analogous analyses of the *UBC4* and *nes* gene segments supports this assumption. As one example, all three markers indicate substantially higher genetic variation in the R-strain than C-strain by both haplotype diversity and nucleotide diversity metrics (Fig 6). This suggests that the R-strain may be more closely related to the ancestral population of the two strains, which agrees with the extrapolations from rooted phylogenetic tree analyses that consistently show the C-strain arising as a branch of the R-strain (Figs 2 and 3).

There is speculation based on molecular clock calculations of strain-specific mitochondrial lineages that the strains first diverged as much as several million years ago [60,61]. However, the relationship between the divergence of these mitochondrial haplotypes and the origins of the current FAW host strains is unclear, particularly since the primary phenotype associated with the strains involve plant hosts that only became domesticated in the Western Hemisphere about 10,000 years ago, *i.e.*, corn [62] and sorghum [63], with corn presumed to have an outsized impact because of its prevalence. It is unknown what phenotypes, if any, distinguished the strain-specific mitochondrial lineages prior to the establishment of the host plants currently associated with FAW. Because our markers are defined by their ability to differentiate FAW populations by host plant, we believe the observed phylogenetic patterns primarily reflect the impact of modern corn agriculture on the evolution of the species. Specifically, corn production in the Western Hemisphere is dominated by extensive monoculture acreages that are systematically treated with pesticides. This subjects the C-strain population to conditions conducive to rapid expansion followed by treatment-induced bottlenecks, a boom-bust cycle expected to substantially alter genetic variation, and one that is unlikely to be experienced by most of the R-strain population. Such large differences in selection are likely to dominate the divergence of the two strains.

Population divergence as measured by *Fst* and *Dxy* are generally consistent with these observed patterns and provide additional insights into how the two strains are evolving. For all three markers, *Dxy* values for comparisons between CS populations (CS-CS) are consistently lower than those calculated for RS-RS and CS-RS comparisons (Fig 7C), and the C-strain also had low *Hd* and *π* compared to the R-strain (Fig 6). These patterns could arise from the C-strain undergoing repeated demographic bottlenecks that reduce overall diversity combined with heightened selection pressures that increase the number of fixed alleles within the strain. Such events would tend to increase *Fst* in CS-RS comparisons to a much larger degree than *Dxy*. While these proposed interactions are clearly speculative, they suggest that the phylogenetic patterns and genetic variation metrics observed could be explained by the association of the C-strain to corn being the primary driver of strain divergence in the Western‌‌ Hemisphere.

**Fig 9:**
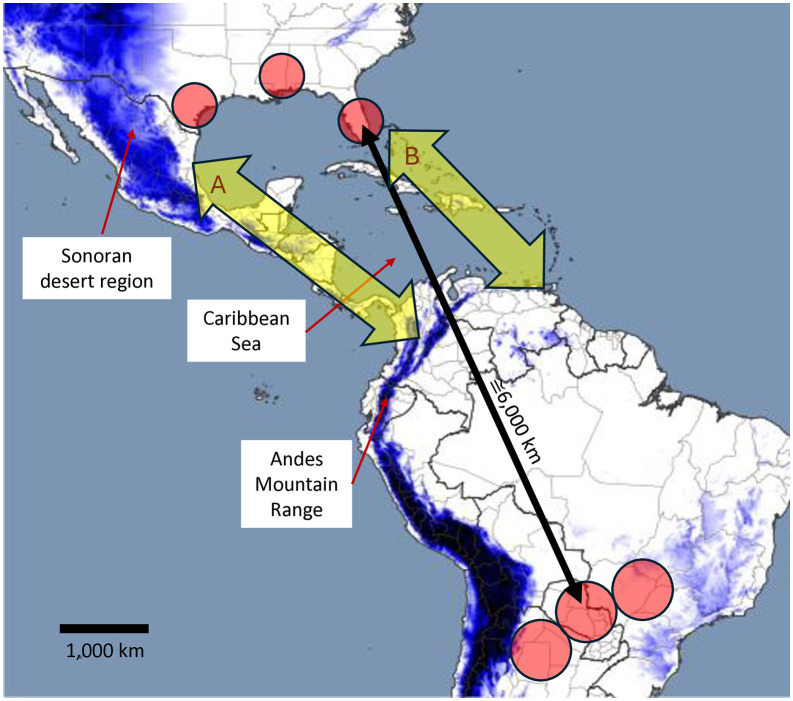
Map FAW collection sites displaying their approximate geographical distance and the location of potential physical barriers to migration between North America and South America. Yellow arrows denote possible migratory pathways between the American continents; A, the Central America land bridge and B, island hopping across the Caribbean islands. Purple areas denote higher elevations. The map was generated using QGIS version 2.18.2 (Open Source Geospatial Foundation). Digital elevation data were downloaded from the open source database https://viewfinderpanoramas.org/ and processed by QGIS.

The collection sites in NA are separated from those in SA by thousands of kilometers and include physical barriers to migration such as the Andes Mountain Range, the Caribbean Sea, and the Mexican Sonoran Desert region (Fig 9). The within strain analyses (CS-CS and RS-RS) compared populations from NA and SA, while the between strain analyses compared populations within the two Americas as well as between the continents, providing an opportunity to quantify the relative contributions of the host strains and geographical separation on FAW genetic variation. We found no indication of geographical population structure from the *Fst* and *Dxy* measures (Fig 8). These results disagree with previously described regional differences in the frequencies of certain mitochondrial haplotypes that persisted over many years, which included differences between some NA and SA populations [64–66]. The mitochondrial haplotype results were interpreted as evidence for significant limitations in gene flow between FAW populations in NA and SA, which was consistent with evidence of restricted FAW exchanges across the two most obvious potential migratory conduits between the Americas, the Caribbean island chain [67] and the Central America land bridge [68,69]. These observations appear to indicate sufficient genetic exchanges between hemispheric populations to make detecting reproducible genetic structure at the nuclear genome level difficult, but not enough to compromise regional mitochondrial haplotype frequency differences that have persisted for multiple decades. The locations and magnitude of these genetic interactions between the American continents remain undefined.

In summary, the identification and characterization of two new strain markers provide an important genetic resource for the investigation of FAW population genetics and the behaviors of the two host strains. This will be particularly relevant in the Eastern Hemisphere where strain identification is less certain, with studies currently underway to exploit these new markers in Africa and Asia. The data from these new loci support conclusions previously based on limited genetic data concerning the primacy of the *Z*-chromosome in strain identity, the strain divergence of the *Z*-chromosome as a single genetic unit, and the differences in the continuation evolution of the two strains as observed by several genetic variation metrics. These markers will facilitate further studies on the population behavior of FAW in the Western Hemisphere and should further advance our understanding of what Western Hemisphere FAW populations are now present in the Eastern Hemisphere and perhaps localize from where they originated.

## Supporting information

S1 Fig DNA sequences for the UBCie695, TpiEI194, and nesEI533 fragments. (TIF)
