# Supplementary figures and images for: Identification and characterization of two new markers for differentiating fall armyworm strains across the Western Hemisphere
Source: PLoS One. 2026 Jun 2;21(6):e0350388. doi: 10.1371/journal.pone.0350388 (PMC13229305; doi:10.1371/journal.pone.0350388)

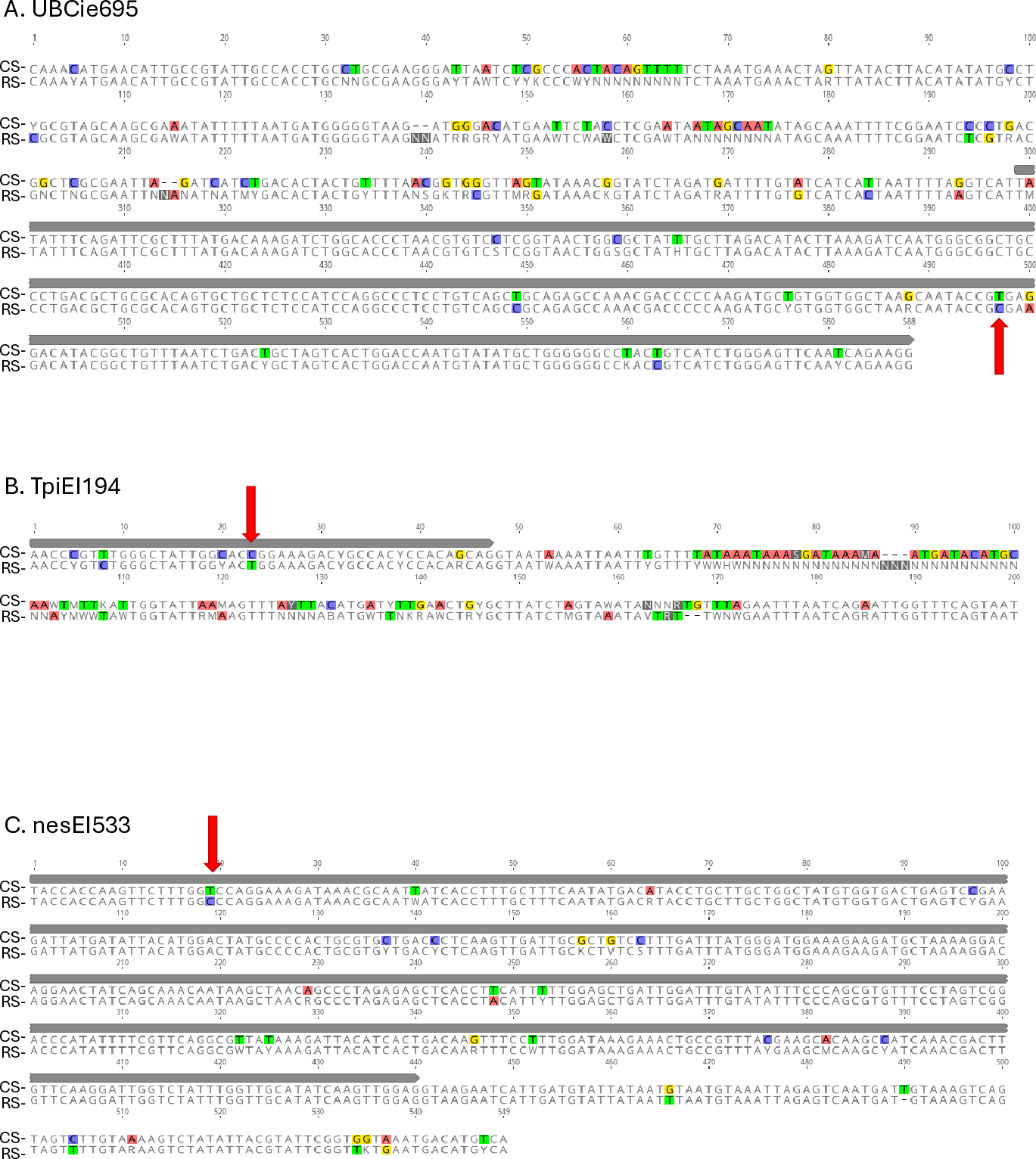

Supplement: S1 Fig — (TIF) [file pone.0350388.s001.tif]
